# Fibroblast Growth Factor 21-Null Mice Do Not Exhibit an Impaired Response to Fasting

**DOI:** 10.3389/fendo.2016.00077

**Published:** 2016-06-30

**Authors:** Patrick Joseph Antonellis, Meghan Patricia Hayes, Andrew Charles Adams

**Affiliations:** ^1^Lilly Research Laboratories, Lilly Corporate Center, Indianapolis, IN, USA

**Keywords:** FGF21, KLB, metabolism, fasting, gluconeogenesis

## Abstract

Fibroblast growth factor 21 (FGF21) is a pleotropic metabolic regulator, expression of which is elevated during fasting. To this end, the precise role played by FGF21 in the biology of fasting has been the subject of several recent studies, which have demonstrated contributions to the regulation of both lipid and carbohydrate metabolism. In the present study, we compared wild-type (WT) and FGF21-null (FGF21KO) mice, demonstrating that, despite the significant induction of FGF21 during fasting in the WT animals, our strain of FGF21-null mice exhibits only limited impairments in their adaptation to nutrient deprivation. Specifically, fasted FGF21KO mice display a mild attenuation of gluconeogenic transcriptional induction in the liver accompanied by partially blunted glucose production in response to a pyruvate challenge. Furthermore, FGF21KO mice displayed only minor impairments in lipid metabolism in the fasted state, limited to accumulation of hepatic triglycerides and a reduction in expression of genes associated with fatty acid oxidation. To address the possibility of compensation to germline deletion of FGF21, we further interrogated the role of endogenous FGF21 *via* acute pharmacological blockade of FGF21 signaling. At the transcriptional level, we show that FGF21 signaling is required for full induction of gluconeogenic and oxidative genes in the liver. However, corroborating our findings in FGF21KO mice, pharmacological blockade of the FGF21 axis did not profoundly disrupt the physiological response to fasting. Taken as a whole, these data demonstrate that, while FGF21 is partially required for appropriate gene expression during the fed to fasted transition, its absence does not significantly impact the downstream physiology of the fasted state.

## Introduction

Fibroblast growth factor 21 (FGF21) is a member of the endocrine subgroup of the FGF family, comprised of FGF19 (FGF15 in mice), FGF21, and FGF23. Unlike the canonical FGFs, members of this family lack a heparin-binding domain, which allows them to enter the circulation (1). Recently, it was demonstrated, through tissue-specific deletion, that the primary source of circulating FGF21 is the liver (2). The systemic physiological effects of FGF21 are mediated by its binding to a receptor complex composed of FGF receptor 1 (FGFR1) and the cofactor protein β-klotho (KLB) (3). While FGFR1 is broadly distributed, KLB is mainly expressed in metabolically active tissues, such as liver, adipose, and pancreas. Thus, it is the expression of KLB, not FGFR1, which restricts the tissues FGF21 can act upon (4, 5).

Acute FGF21 administration in mice leads to activation of the FGF signaling pathway in both liver and adipose (6). However, deletion of FGFR1 or KLB, specifically in adipose, demonstrates that adipose tissue activation is intrinsically required for FGF21’s pharmacological effects (7, 8). In addition to these peripheral sites of action, it has recently been proposed that FGF21 may act centrally (9). Concordant with this assertion, expression of KLB has been reported in discrete areas of the hypothalamus, deletion of which has been reported to abrogate the metabolic effects of exogenous FGF21 administration (10). It has also been demonstrated that central FGF21 activates the sympathetic nervous system and that intact sympathetic tone is required to mediate its effects at the periphery (11, 12).

In the liver, FGF21 expression primarily resides downstream of the transcription factor, peroxisome proliferator-activated receptor alpha (PPARα) (13). PPARα and, subsequently, FGF21 are induced in states that demand enhanced hepatic lipid oxidation, such as fasting and ketogenic diet feeding (14, 15). Interestingly, FGF21-null (FGF21KO) mice have previously been reported to exhibit impairments in glucose and lipid metabolism (16). Furthermore, in response to feeding of a ketogenic diet, both global deletion and liver-specific FGF21 knockdown lead to hepatic triglyceride accumulation, accompanied by an attenuation of hepatic oxidative gene expression (14, 17). In addition to defects in lipid metabolism, FGF21KO mice also exhibit enhanced adipose mass coupled with the development of larger adipocytes (17–19). Conversely, FGF21 transgenic mice (FGF21Tg) are leaner than their WT counterparts, with a concomitant increase in hepatic fatty acid oxidation (15, 16). In agreement with the phenotype of FGF21Tg animals, pharmacologic treatment with FGF21 leads to a significant decrease in body mass in preclinical models (20, 21). This reduction in body mass is primarily due to decreased adipose mass, thought to be driven in large part by significantly increased energy expenditure and a reduction in respiratory exchange ratio (RER), which when taken together are suggestive of a state of elevated lipid oxidation (20).

In the present study, we utilize both genetic and pharmacological approaches to assess the physiological role of FGF21 during fasting. We demonstrate that, while our own strain of FGF21KO mice do exhibit impairments in the transcriptional regulation of pathways involved in lipid and carbohydrate metabolism during fasting, these defects had little in the way of physiological consequences. To further interrogate the physiological role of FGF21 during fasting and to address the potential for confounding developmental compensation in the FGF21KO mice, we sought to directly inhibit endogenous FGF21 signaling. To do so, we utilized a variant of the wild-type human FGF21 protein lacking 17 N-terminal amino acid residues, termed ΔN17, which we have previously shown blocks FGF21 action *in vivo* (22). In agreement with the effects observed in FGF21KO mice, we found that treatment with ΔN17 either during the early fed to fasted transition or after a prolonged fast did significantly impact the transcriptional response, yet led to only minor physiological effects. Taken as a whole, these studies demonstrate that, while manipulation of the FGF21 axis can modify the transcriptional response to caloric deprivation, FGF21 is not explicitly required for an appropriate, integrated, physiological response to fasting.

## Materials and Methods

### Proteins

Human FGF21ΔN17 was generated as previously described (23).

### Animals

All animals were individually housed in a temperature-controlled (24°C) facility with 12 h/12 h light/dark cycle. Animal protocols in this study were approved by the Eli Lilly and Co., Animal Use and Care Committee (Protocol No. 13-030).

### Comparison of WT and FGF21KO Animals in the Fed and Fasted States

Male C57Bl/6J and FGF21 knockout (FGF21KO) mice (Taconic Farms) were maintained on a standard chow diet consisting of 13% fat, 67% carbohydrate, and 20% protein caloric content (2014; Harlan Teklad, Madison, WI, USA) and had free access to food and water before randomization by weight. A cohort of mice from each genotype had their food removed at the beginning of the light cycle and were sacrificed following a 24 h fast. A separate cohort from each genotype was allowed *ad libitum* access to food and sacrificed at the end of the dark cycle. Prior to sacrifice, blood glucose levels were assessed using handheld glucometers (Accu-Chek Aviva; Roche Diagnostics, Indianapolis, IN, USA). Following sacrifice, liver and epididymal white adipose tissue (WAT) samples were rapidly dissected and flash frozen in liquid nitrogen. Whole blood was collected in EDTA coated tubes, centrifuged, and plasma collected for analysis. Each cohort consisted of at least six mice.

### Acute Treatment of WT Animals

Male C57Bl/6J mice (Taconic Farms) 11–12 weeks of age were maintained on a standard chow diet (2014; Harlan Teklad). At the beginning of the light cycle, mice had their food removed and dosed with either 5 mg/kg of ΔN17 or vehicle *via* intraperitoneal (IP) injection. Number of injections and duration of study were as indicated. At the indicated time points, blood glucose was measured using handheld glucometers (Accu-Chek Aviva; Roche Diagnostics). At the end of each study, mice were sacrificed, and liver and WAT samples were rapidly dissected and flash frozen in liquid nitrogen. Whole blood was collected in EDTA-coated tubes, centrifuged, and plasma removed for analysis. Six mice were used for each treatment group.

### Pyruvate Tolerance Test

Mice were fasted for the indicated amount of time. Following the fast, animals were administered 2 g/kg sodium pyruvate *via* IP injection. Blood glucose was measured at the indicated time points using handheld glucometers (Accu-Chek Aviva; Roche Diagnostics).

### Metabolites and Hormones

Plasma triglycerides and free fatty acids were measured using a Hitachi 912 Clinical Chemistry analyzer (Roche Diagnostics). To measure liver triglyceride content, a 10% tissue homogenate was prepared and analyzed using a Hitachi 912 Clinical Chemistry analyzer. Circulating levels of insulin (Ultra Sensitive Mouse Insulin ELISA; CrystalChem, Downers Grove, IL, USA) and FGF21 (Mouse/Rat FGF21 Quantikine ELISA; R&D Systems, Minneapolis, MN, USA) were determined by ELISA assay. Colorimetric assays were used to determine plasma βHB (β-hydroxybutyrate LiquiColor; Stanbio Laboratories, Boerne, TX, USA) and liver glycogen content (Glycogen Assay Kit; Sigma-Aldrich, St. Louis, MO, USA).

### Protein Measurements

Protein was extracted from tissue using mammalian protein extraction reagent (M-PER) (Pierce, Rockford, IL, USA) containing 1× halt protease and phosphatase inhibitor cocktail (Pierce). FGF21 protein concentration was determined by ELISA (Mouse/Rat FGF21 Quantikine ELISA; R&D Systems). The average minimum detectable dose of the assay is 3.81 pg/mL, and all samples measured fell within the linear range of 31.3–2000 pg/mL. Total and phosphorylated hormone-sensitive lipase (HSL) was determined by Western blot. Briefly, WAT protein extracts were separated by SDS-PAGE and transferred to nitrocellulose membranes, which were probed using anti-rabbit antibodies to total and phosphorylated HSL (Cell Signaling Technologies, Danvers, MA, USA).

### RNA Isolation, RT, and Real-time Quantitative PCR

RNA was isolated from tissues using TRIzol reagent (Invitrogen, Carlsbad, CA, USA) and the RNeasy lipid mini kit (Qiagen, Venlo, Netherlands) and then reverse transcribed into cDNA using the Quantitect Reverse Transcription Kit (Qiagen). Reactions were performed on an Applied Biosystems QuantStudio 7 Real-Time PCR System (Applied Biosystems, South San Francisco, CA, USA), CT values were normalized to β-actin, and relative expression was calculated by the ΔΔCT method. Fold change was calculated by normalizing relative expression to the proper control, WT-fed or PBS-treated animals. Assays-on-Demand Gene expression products (Applied Biosystems) were as follows: CPT1a Mm01231183-m1; ACADS Mm00431617_m1; ACADM Mm01323360_g1; ACADL Mm00599660_m1; ACADVL Mm0044293_m1; HADH Mm00492535_m1; CD36 Mm01135198_m1; G6PC Mm00839363-m1; PCK1 Mm01247058-m1; HMGCS2 Mm00550050_m1; BDH1 Mm00558330_m1; PGC1α Mm01208835-m1; CIDEA Mm00432554_m1; UCP1 Mm01244861_m1; FASN Mm00662319_m1; HSL Mm00495359_m1; ATGL Mm00503040_m1; LPL Mm00434770_m1; and FGF21 Mm00840165_g1.

### Statistical Analysis

Data are presented as mean ± SEM. Statistical analysis was performed using two-way ANOVA or one-way ANOVA followed by Dunnett’s multiple comparisons test or *T*-test where appropriate. Differences were considered significant when *P* < 0.05.

## Results

### FGF21KO Mice Exhibit Modest Alterations in Lipid and Carbohydrate Metabolism during a Fast

**Figure 1:**
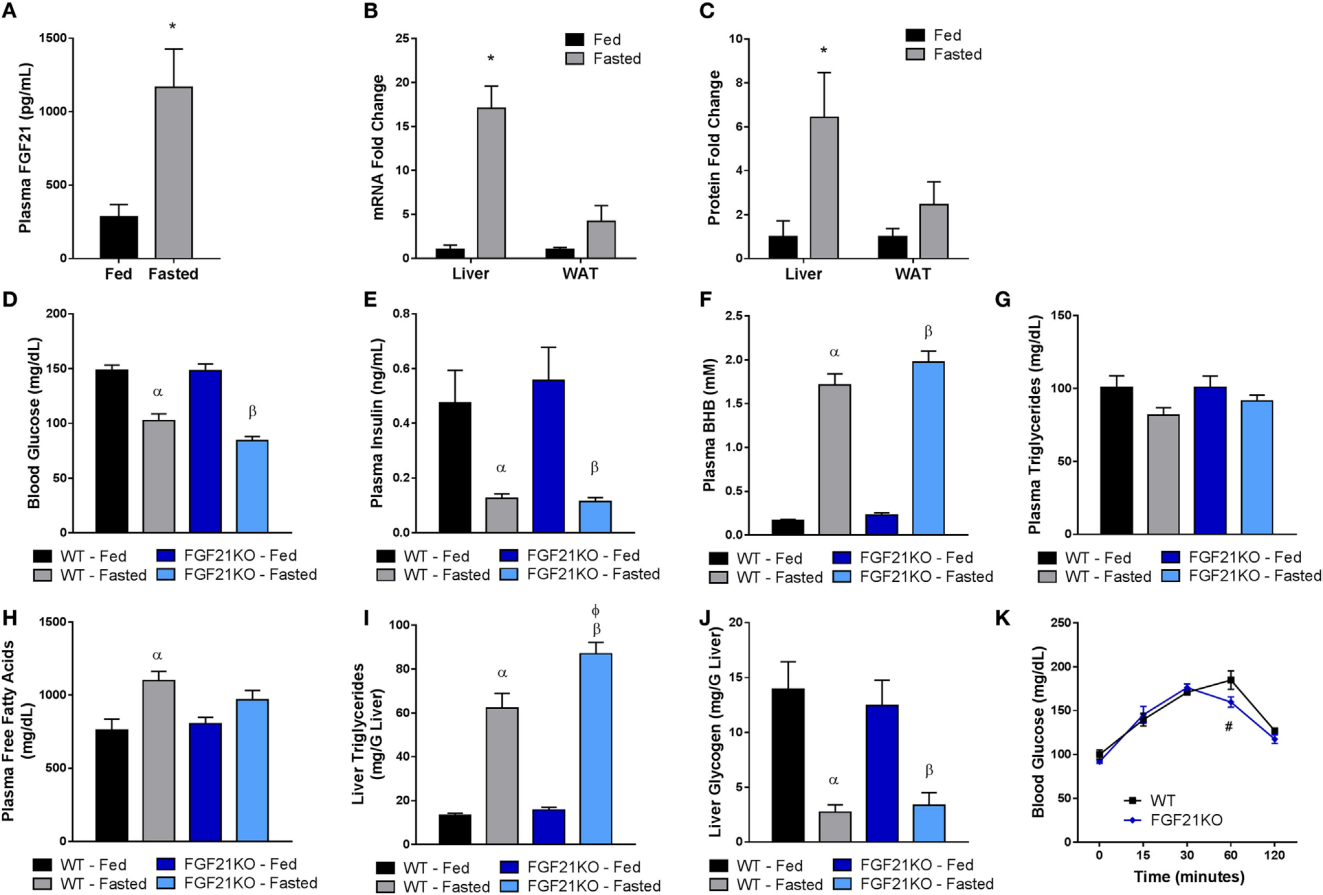
**FGF21 is primarily expressed in the liver and is dispensable for the physiological response to fasting**. WT and FGF21KO animals were either allowed *ad libidum* access to food (fed) or subjected to a 24-h fast (fasted). **(A)** Plasma FGF21 levels were measured in WT animals. **(B)** FGF21 mRNA and **(C)** protein expression were measured in liver and adipose tissue from WT animals. **(D)** Blood glucose was assessed *via* glucometer in each cohort. Plasma was collected from WT and FGF21KO animals and used to determine circulating levels of **(E)** insulin, **(F)** βHB, **(G)** triglycerides, and **(H)** free fatty acids. The concentration of **(I)** triglycerides and **(J)** glycogen was measured in the livers of WT and FGF21KO animals. **(K)** A pyruvate tolerance test was performed on a separate cohort of WT and FGF21KO animals by administration of 2 g/kg sodium pyruvate following a 22-h fast. *p < 0.05 vs. fed; α, *p* < 0.05 vs. WT Fed; β, *p* < 0.05 vs. FGF21KO fed; φ, *p* < 0.05 vs. WT fasted; #, *p* < 0.05 vs. WT. No statistical difference was observed in fed measurements between genotypes.

To assess the role of FGF21 during the adaptive response to fasting, we compared cohorts of WT and FGF21KO mice in both the fed state and following a 24-h fast. We found that increased plasma levels of FGF21 during fasting (Figure 1A) correlated with a significant elevation in FGF21 mRNA (Figure 1B) and protein (Figure 1C) in the liver, but not adipose tissue (Figures 1B,C), consistent with recent findings (2). Both WT and FGF21KO animals exhibited expected reductions in blood glucose (Figure 1D) and insulin (Figure 1E). However, while FGF21KO animals did appear to have mildly reduced blood glucose levels compared to WT mice in the fasted state, this effect was not statistically significant (Figure 1D).

**Figure 2:**
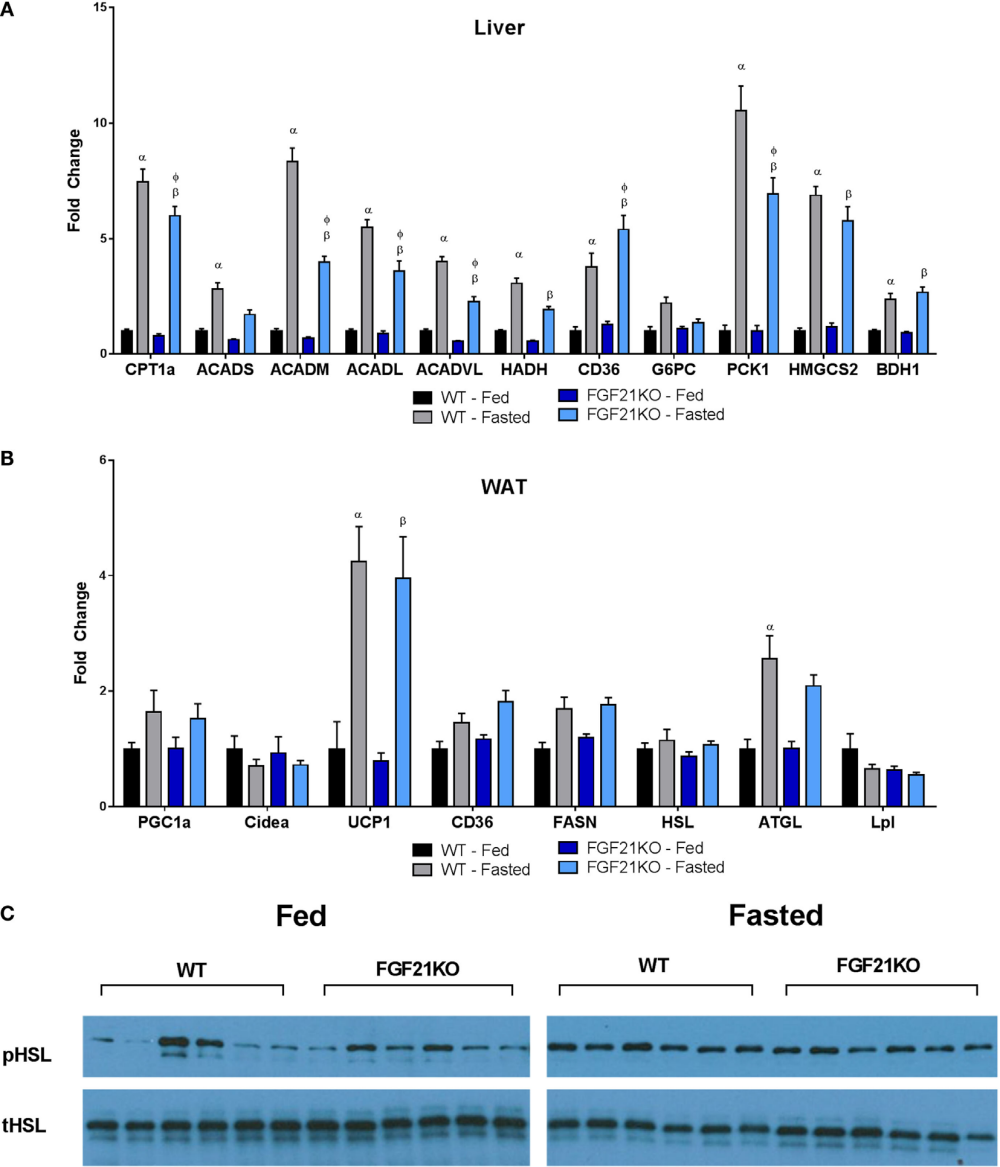
**Oxidative and gluconeogenic gene expression is attenuated in the liver, while lipase gene expression and activity in WAT remains unaltered in fasted FGF21KO mice**. **(A)** In the liver, expressions of genes associated with fatty acid oxidation (CPT1a, ACADS, ACADM, ACADL, ACADVL, HADH), fatty acid import (CD36), gluconeogenesis (G6PC, PCK1), and ketone body production (HMGCS2, BDH1) were measured. **(B)** Expressions of genes associated with lipolysis (HSL, ATGL, LPL), fatty acid synthesis (FASN), fatty acid import (CD36), and thermogenesis (CIDEA, UCP1) were measured. In adipose tissue: α, *p* < 0.05 vs. WT Fed; β, *p* < 0.05 vs. FGF21KO fed; φ, *p* < 0.05 vs. WT fasted. No statistical differences were observed between genotypes in fed expression of any gene measured. **(C)** The amount of total and phosphorylated HSL was assessed by Western blot in WAT of WT and FGF21KO animals in both the fed and fasted state.

It has been demonstrated that FGF21 is important for the induction of ketosis when animals are maintained on a ketogenic diet (14, 16). However, consistent with previous studies (17), our strain of FGF21KO animals fed on a chow diet did not show impaired βHB production in the fasted state (Figure 1F). Nor were circulating triglycerides altered in either genotype (Figure 1G). Interestingly, plasma free fatty acids were elevated in fasted WT, but not FGF21KO animals (Figure 1H). Therefore, as FGF21 has been implicated in the regulation of lipolysis (18), we investigated whether altered lipase activity in the WAT of the FGF21KO animals was responsible for this discrepancy. We found no differences in the expression of HSL or lipoprotein lipase (LPL); however, while induction of adipose triglyceride lipase (ATGL) in the fasted state did not reach significance in FGF21KO mice, it did trend upward to a similar degree as observed in the WT animals (Figure 2B). Furthermore, during fasting, we observed equivalent phosphorylation of HSL in adipose tissue of WT and FGF21KO animals (Figure 2C). In addition to mediators of lipolysis, we assessed the known FGF21 target genes peroxisome proliferator-activated receptor gamma coactivator 1-alpha (PGC1α) and uncoupling protein 1 (UCP1) and found no differences in their expression between the genotypes (Figure 2B).

While both WT and FGF21KO mice exhibited elevated hepatic triglyceride content following a fast, FGF21KO animals accumulated significantly more than their WT counterparts (Figure 1I). As we found no evidence of enhanced lipolysis in adipose tissue, we next examined the expression of genes associated with fatty acid import and oxidation in the liver. Importantly, we found that the induction of genes involved in fatty acid oxidation (carnitine palmitoyltransferase 1A, CPT1a; acyl-Coenzyme A dehydrogenase, short chain, ACADs; acyl-Coenzyme A dehydrogenase, medium chain, ACADM; acyl-Coenzyme A dehydrogenase, long chain, ACADL; acyl-Coenzyme A dehydrogenase, very long chain, ACADVL) were attenuated in FGF21KO animals (Figure 2A). Additionally, FGF21KO animals exhibited a significantly greater increase in expression of the fatty acid transporter, CD36, in the fasted state (Figure 2A).

Next, we sought to determine the impact of global FGF21 deletion on carbohydrate metabolism during fasting. We found that, while there was no difference in glucose-6-phosphatase (G6PC) expression, induction of phosphoenolpyruvate carboxykinase 1 (PCK1) during fasting was significantly attenuated in FGF21KO mice (Figure 2A). To assess whether the observed attenuation in PCK1 gene expression in FGF21KO mice impacted gluconeogenic capacity, we conducted a pyruvate tolerance test (PTT). To our surprise, we found that FGF21KO mice exhibited only a modest reduction in gluconeogenesis following a pyruvate challenge (Figure 1K). This minor effect on carbohydrate metabolism was reflected in maintenance of normal hepatic glycogen content in the FGF21KO animals (Figure 1J).

### Pharmacological Inhibition of Endogenous FGF21 Signaling Has No Effect on Lipid and Carbohydrate Utilization during the Early Fed to Fasted Transition

**Figure 3:**
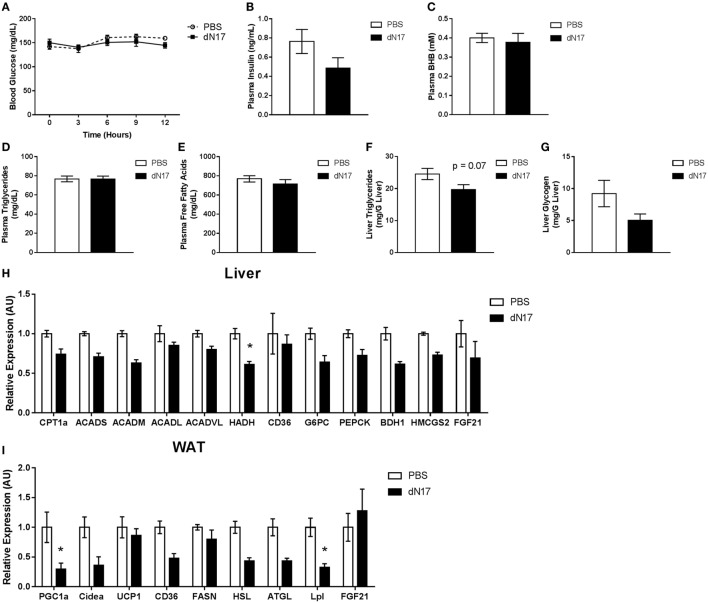
**Endogenous FGF21 is not required for proper lipid and carbohydrate metabolism during the fed to fasted transition**. At the beginning of the light cycle, WT animals had their food removed and were administered 5 mg/kg ΔN17 once every 3 h for 12 h. **(A)** Blood glucose measurements were taken *via* glucometer at the specified time points. Circulating levels of **(B)** insulin, **(C)** βHB, **(D)** triglycerides, and **(E)** free fatty acids were determined from plasma samples collected at the end of the study. Liver samples were taken at the end of the study and used to determine the concentrations of **(F)** triglycerides and **(G)** glycogen. **(H)** In the liver, expression of FGF21 as well as genes associated with fatty acid oxidation (CPT1a, ACADS, ACADM, ACADL, ACADVL, HADH), fatty acid import (CD36), gluconeogenesis (G6PC, PCK1), and ketone body production (HMGCS2, BDH1) were measured. **(I)** Expression of FGF21 in addition to genes associated with lipolysis (HSL, ATGL, LPL), fatty acid synthesis (FASN), fatty acid import (CD36), and thermogenesis (CIDEA, UCP1) were measured in adipose tissue. *p < 0.05 vs. PBS.

To investigate the potential role of endogenous FGF21 during the transition from the fed to fasted state and to mitigate potential concerns regarding developmental compensation to germline deletion of FGF21, we subjected WT animals to a time course of FGF21–ΔN17 (ΔN17) treatment. Since ΔN17 functions as an antagonist of FGF21 action *in vivo* (22), pharmacological administration of the protein allows for acute blockade of the FGF21 axis. At the beginning of the light cycle, animals had their food removed, then were dosed with ΔN17 every 3 h for 12 h. During this initial transition period, treatment with ΔN17 had no significant effect on blood glucose (Figure 3A) or plasma insulin levels (Figure 3B). Additionally, there was no effect on circulating levels of βHB (Figure 3C), triglycerides (Figure 3D), free fatty acids (Figure 3E), hepatic triglyceride (Figure 3F), or liver glycogen content (Figure 3G). When gene expression was assessed in the liver, we found that of the genes involved in fatty acid oxidation (CPT1a, ACADS, ACADM, ACADVL, HADH), only HADH was significantly altered by ΔN17 treatment (Figure 3H). Similarly, genes related to gluconeogenesis (G6PC, PCK1) and ketone body production (3-hydroxybutyrate dehydrogenase, type 1, BDH1; 3-hydroxy-3-methylglutaryl-CoA synthase 2, HMGCS2) were unaffected (Figure 3H). Somewhat surprisingly, in the WAT of animals treated with ΔN17, we did observe reduced expression of PGC1α (Figure 3I). These data lead us to conclude that endogenous FGF21 has a limited role during early fasting, supportive of earlier studies, which demonstrated that full induction of hepatic and serum FGF21 requires prolonged fasting (14, 15).

### Interruption of FGF21 Signaling during Prolonged Fasting Does Not Significantly Impact Lipid or Carbohydrate Metabolism

**Figure 4:**
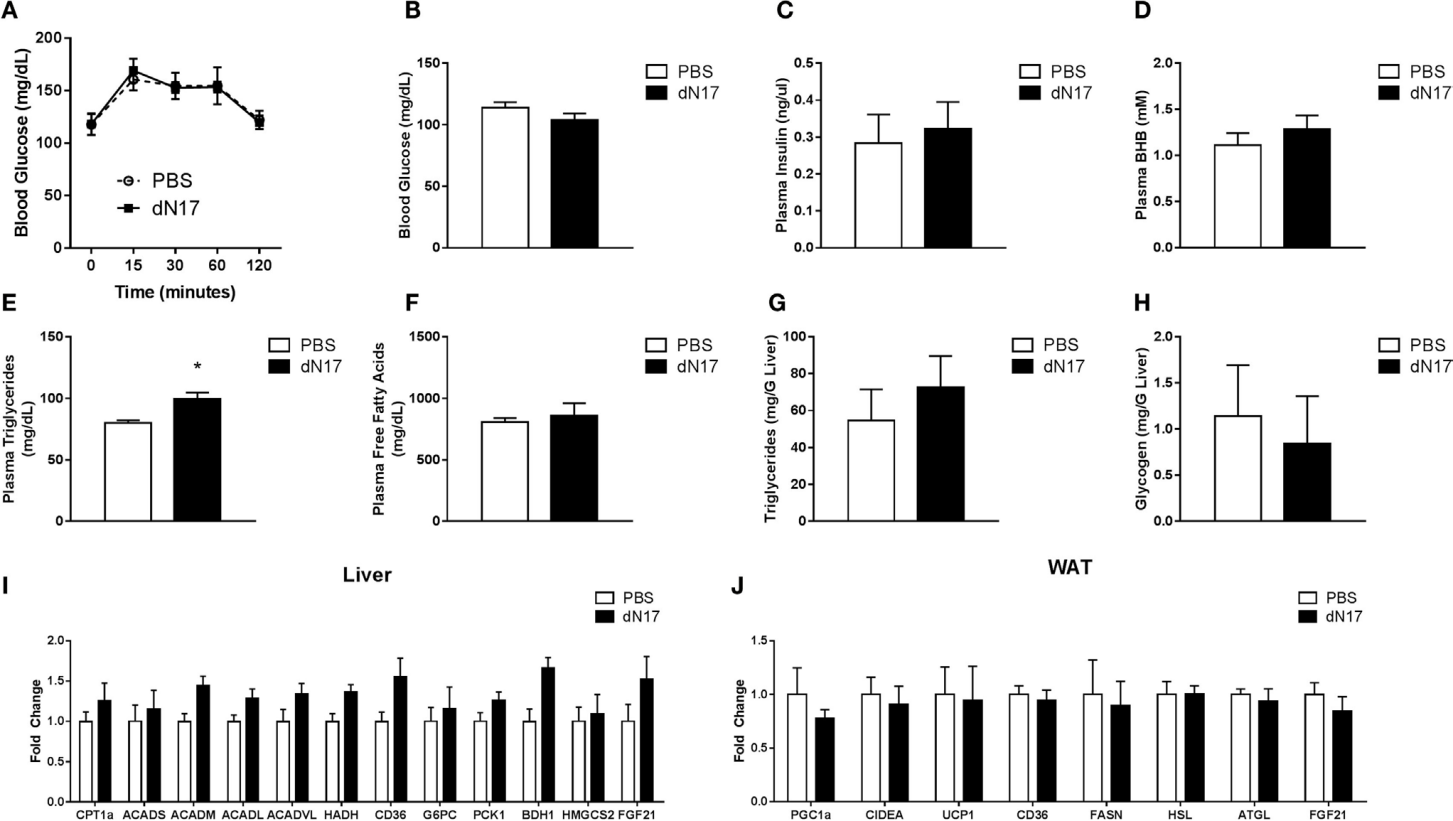
**Blockade of FGF21 signaling during late fasting does not impair the fasted response**. WT mice were fasted for 21 h, administered 5 mg/kg ΔN17, and then subjected to a PTT 1 h later by administration of 2 g/kg sodium pyruvate. **(A)** Blood glucose was measured at the specified time points *via* glucometer. A separate cohort of WT mice was fasted for 23 h, administered 5 mg/kg ΔN17, and then sacrificed 1 h later. **(B)** Blood glucose was assessed *via* glucometer. Plasma samples were collected and used to determine circulating levels of **(C)** insulin, **(D)** βHB, **(E)** triglycerides, and **(F)** free fatty acids. Hepatic **(G)** Triglyceride and **(H)** glycogen content was measured. **(I)** In the liver, expression of FGF21 as well as genes associated with fatty acid oxidation (CPT1a, ACADS, ACADM, ACADL, ACADVL, HADH), fatty acid import (CD36), gluconeogenesis (G6PC, PCK1), and ketone body production (HMGCS2, BDH1) were measured. **(J)** Expression of FGF21 in addition to genes associated with lipolysis (HSL, ATGL, LPL), fatty acid synthesis (FASN), fatty acid import (CD36), and thermogenesis (CIDEA, UCP1) were measured in adipose tissue. *p < 0.05 vs. PBS.

To assess the role of FGF21 in the regulation of gluconeogenesis during late fasting, WT animals were fasted for 21 h, given a single injection of ΔN17, and then subjected to a PTT. Consistent with our earlier studies in the FGF21KO mice, we found that ΔN17 treatment had no effect on gluconeogenesis following a pyruvate challenge (Figure 4A). To determine whether other discrete aspects of the fasted response were impacted by inhibition of FGF21 signaling, a separate cohort of 23-h fasted mice was treated with a single injection of ΔN17 and sacrificed 1 h later. In agreement with our previous findings, acute treatment with ΔN17 had no significant effect on circulating glucose (Figure 4B), insulin (Figure 4C), βHB (Figure 4D), or free fatty acid (Figure 4F) levels. Mice treated with ΔN17 had significantly elevated plasma triglycerides (Figure 4E); however, both hepatic triglyceride (Figure 4G) and glycogen (Figure 4H) content were unchanged by ΔN17 treatment. The gene expression profile in the ΔN17-treated animals exhibited trends reflective of the results observed in the FGF21KO mice; however, none of these differences reached statistical significance in the liver (Figure 4I) or adipose (Figure 4J), suggesting that chronic changes to FGF21 tone may be required to observe the full magnitude of its effects.

## Discussion

Despite significant study, including the generation of three separate FGF21-null models (16–18), the physiological role of FGF21 remains uncertain. Broadly, it has been proposed that a primary role of FGF21 is to coordinate lipid and carbohydrate metabolism during nutrient deprivation. In support of this view, FGF21 has been shown to be induced by nutrient-deficient states such as prolonged fasting as well as during exposure to ketogenic (17) and protein-deficient diets (24–26). In the present study, we investigated the role of FGF21 in the response to fasting through examination of our own strain of FGF21KO animals, in addition to pharmacological inhibition of endogenous FGF21 action.

Congruent with previously published findings (14), we show that, following a 24-h fast, plasma concentrations of FGF21 were indeed significantly elevated in WT mice. This increase in circulating FGF21 corresponded with a significant elevation in FGF21 mRNA and protein in the liver, but not adipose, supportive of a recent report describing the liver as the primary source of endocrine FGF21 (2). We went on to extend these findings by demonstrating that, while FGF21KO mice were able to maintain glycemia in a manner equivalent to their WT counterparts, they did exhibit defects in lipid metabolism during fasting. The elevation of circulating free fatty acids in the fasted state observed in WT mice was abolished in FGF21KO animals despite both genotypes having similar HSL abundance and activity in WAT. Interestingly, in addition to reduced serum FFA levels, the FGF21-null animals also exhibited elevated hepatic triglyceride accumulation in the fasted state. When hepatic gene expression was assessed, we found attenuation in the induction of genes involved in fatty acid oxidation as well as increased expression of the fatty acid transporter, CD36. Therefore, we suggest that reduced oxidative capacity coupled with increased fatty acid import in the liver may serve to explain both the attenuation of free fatty acids in circulation and the increased hepatic triglyceride content observed in FGF21KO mice during fasting conditions. In support of this conclusion, it has been demonstrated that hepatic CD36 correlates with dyslipidemia in diet-induced obese animals; furthermore, overexpression of CD36 in the liver leads to increased fatty acid uptake and triglyceride storage in the liver (27). It has also been shown that reducing fatty acid oxidation through the use of a specific inhibitor of liver CPT1 causes hepatic triglyceride accumulation (28). Thus, the coupling of these two mechanisms would serve as a mechanistic basis for the impaired lipid homeostasis observed in our strain of FGF21KO mice.

The primary manner in which FGF21 is thought to regulate glucose metabolism is *via* modulation of hepatic gluconeogenesis (29). In support of this contention, FGF21-null animals have been reported to exhibit attenuated hepatic gluconeogenic gene expression in the fasted state (16). Furthermore, it has also been shown that this transcriptional defect translates to a functional impairment in gluconeogenesis following a fast (30). However, here, we show that despite attenuation in gluconeogenic gene expression in the liver, our own strain of FGF21KO animals had no significant impairment of gluconeogenesis, exhibiting nearly identical endogenous glucose production as WT mice. These results are in agreement with the phenotype reported in a third strain of FGF21KO mice (31).

Given the discordance between the three strains of FGF21KO mice and to avoid potential confounding effects of compensation due to germline deletion, we further interrogated the role of the FGF21 axis in the fasted state, pharmacologically. Specifically, we inhibited endogenous FGF21 through the use of a truncated form of the protein (ΔN17) during both the early and late phases of the fed to fasted transition. Treatment with ΔN17 had little effect on early fasting, which given the short nature of the fast (12 h) and that animals would not normally be eating much during this time, is not wholly unexpected. However, in 24-h-fasted animals, we found that interruption of FGF21 signaling led to little more than a blunting of the transcriptional response to fasting. Indeed, in spite of a diminished transcriptional response, ΔN17 treatment had no effect on hepatic gluconeogenesis in response to a pyruvate challenge nor did it significantly alter systemic lipid homeostasis. These results were reminiscent of the physiological effects observed in two of the three strains of FGF21KO mice; however, they did not fully recapitulate the phenotype observed in those animals. It is possible that transcriptional changes during later stages of fasting are required for FGF21 to exert its physiological effects; therefore, longer duration of ΔN17 coverage would be required. Alternatively, the nature of endogenous FGF21 action may exist not under the conditions of prolonged fasting, but instead become manifest in a state of true starvation. Thus, longer periods of nutrient deprivation (>24 h) followed by ΔN17 administration may illuminate the biological processes under the control of elevated FGF21 during fasting.

While it appears that FGF21 is required for a full transcriptional response to fasting, the physiological consequences of its loss are extremely limited in nature. Furthermore, those physiological differences we did observe were related specifically to the uptake and subsequent oxidation of hepatic lipids. Taken as a whole, these findings support the view that FGF21 does not play a meaningful role in the mobilization of energy stores during nutrient deprivation, but, instead, acts in an autocrine manner in the fasted state to facilitate hepatic lipid homeostasis. We suggest that, given that the transcriptional changes are consistent in all three lines of FGF21KO mice, the range of physiological responses observed may be due to the environment in which the mice are housed or the variance in the specific experimental conditions of the studies.

## Author Contributions

PA, MH, and AA designed and executed the studies and also analyzed the data and wrote the manuscript.

## Conflict of Interest Statement

All studies were conducted by Eli Lilly and Company. All authors were employees of Eli Lilly and Company.
